# The effect of inter-pregnancy interval on stillbirth in urban South Ethiopia: a community-based prospective cohort study

**DOI:** 10.1186/s12884-021-04325-z

**Published:** 2021-12-29

**Authors:** Belayneh Hamdela Jena, Gashaw Andargie Biks, Yigzaw Kebede Gete, Kassahun Alemu Gelaye

**Affiliations:** 1 Department of Epidemiology and Biostatistics, Institute of Public Health, College of Medicine and Health Sciences, University of Gondar, Gondar, Ethiopia; 2 Department of Public Health, College of Medicine and Health Sciences, Wachemo University, Hossana, Ethiopia; 3 Department of Health System and Policy, Institute of Public Health, College of Medicine and Health Sciences, University of Gondar, Gondar, Ethiopia

**Keywords:** Stillbirth, Inter-pregnancy interval, Pregnant women, Cohort, Urban South Ethiopia

## Abstract

**Background:**

Stillbirth is an invisible and poorly understood adverse pregnancy outcome that remains a challenge in clinical practice in low-resource settings. It is also a key concern in Ethiopia where more than half of pregnancies occur shortly after preceding childbirth. Whether the interval between pregnancies has an effect on stillbirth or not is unclear. Therefore, we aimed to assess the effect of inter-pregnancy interval on stillbirth in urban South Ethiopia.

**Methods:**

A community-based prospective cohort study was conducted among 2578 pregnant women and followed until delivery. Baseline data were collected at the household level during registration and enrolment. End-line data were collected from hospitals during delivery. Exposed groups were pregnant women with inter-pregnancy intervals < 18 months and 18–23 months. Unexposed group contains women with inter-pregnancy intervals 24–60 months. A generalized linear model for binary outcome was applied, using R version 4.0.5 software. Relative risk (RR) was used to estimate the effect size with a 95% confidence level. Attributable fraction (AF) and population attributable fraction (PAF) were used to report the public health impact of exposure.

**Results:**

The overall incidence of stillbirth was 15 per 1000 total births, (95% CI: 11, 20%). However, the incidence was varied across months of inter-pregnancy intervals; 30 (< 18 months), 8 (18–23 months) and 10 (24–60 months) per 1000 total births. The risk of stillbirth was nearly four times (ARR = 3.55, 95%CI: 1.64, 7.68) higher for women with inter-pregnancy interval < 18 months as compared to 24–60 months. This means, about 72% (AF = 72, 95%CI: 39, 87%) of stillbirth among the exposed group (inter-pregnancy interval < 18 months category) and 42% (PAF = 42, 95%CI: 23, 50%) of stillbirth in the study population were attributed to inter-pregnancy interval < 18 months. These could be prevented with an inter-pregnancy interval that is at least 18 months or longer.

**Conclusions:**

Inter-pregnancy interval under 18 months increases the risk of stillbirth in this population in urban South Ethiopia. Interventions targeting factors contributing to short inter-pregnancy intervals could help in reducing the risk of stillbirth. Improving contraceptive utilization in the community could be one of these interventions.

**Supplementary Information:**

The online version contains supplementary material available at 10.1186/s12884-021-04325-z.

## Background

We define stillbirth as a baby born with no signs of life (spontaneous breathing or heartbeat) at or after 28 weeks gestation or a baby weight at birth > 1000 g [1, 2]. It can occur either during prepartum (macerated) or intrapartum (fresh) and has a long-lasting medical, psychological, social and economic impact on the mothers and their families [3–5].

Globally, it is estimated that 2 million stillbirths occur each year. Of these, the vast majorities (84%) occur in low and middle-income countries. More than three quarters (76%) of the estimated stillbirths occurred in Sub-Saharan Africa (42%) and South Asia (34%). Sub-Saharan Africa alone has a stillbirth rate about 7 times that of developed countries (21.7 vs. 3.1 per 1000 births) [6]. Ethiopia, with a stillbirth rate of 24.6 per 1000 births, is among six countries that contribute to 1 million stillbirths across the globe and it is one of the highest in East African countries [6]. A pooled estimate in Ethiopia also indicated a stillbirth rate of 36.9 per 1000 births [7]. As in many Sub-Sahara African countries, stillbirth in Ethiopia is an invisible and poorly understood adverse pregnancy outcome that remains a challenge in clinical practice [8–10]. Usually, it is not counted, registered, publicized for funerals and families could not earn support from local, and social organizations [11]. When it happens during the first pregnancy, people assume that the marriage is unlucky. This results in fear, anxiety and depression for subsequent pregnancies, and affects marital, familial and social relationships of the mother due to repeated blame, stigma and violence [5, 11].

There are numerous risk factors for stillbirth, including maternal age < 25 and ≥ 35 years [12, 13], no education [11, 14, 15], multiparity [14], lower number of antenatal care (ANC) [12, 16], preeclampsia [16, 17], premature rupture of membranes [15], prematurity [13, 17, 18], low birth weight [17, 19, 20], history of perinatal death [16, 21], prolonged labor ≥24 h [20, 22], uterine abruption [17, 20] and birth interval < 24 months [22].

Inter-pregnancy interval (IPI) is defined as a time elapsed from live birth to subsequent conception or a woman’s last menstrual period (LMP) [23]. IPI < 6, < 18 and < 24 months were found to be associated with increased risk of prematurity, low birth weight and small for gestational age [24–26]. Longer IPI (> 59 months) is also related to the three adverse outcomes [25]. Both short (< 6 months) and long (> 60 months) IPIs reported as having no impact on long-term cardiovascular hospitalizations (such as pericarditis, heart failure, rheumatic fever, and ischemic heart disease) of offspring in later life (age up to 18 years) [27].

More than 40 % of stillbirths occur during labor and delivery (intrapartum), suggesting the need for appropriate care and interventions during this time. In 2019, the global average stillbirth rate was declined to 13.9 per 1000 births, a 35% reduction from 2000 (21.4 per 1000 births). The progress in Sub-Saharan Africa has shown to be slowest [6]. In Ethiopia, there was no significant reduction trend in stillbirth for decades, rather it was alarmingly increasing [7]. Some of the reasons for the slowest progress in these regions are poor health infrastructures, lack of skilled health care providers and poor pregnancy, and obstetric care [6, 28].

Despite routine care, stillbirth lacks global attention; neither Millennium nor Sustainable Development Goals considered stillbirth reduction in global targets. Ethiopia has been implementing a global Every Newborn Action Plan since 2014, which is targeted to reduce the stillbirth rate to 12 per 1000 births, by 2030 [29]. In addition to routine maternal health services (such as antenatal and delivery), maternity waiting areas were established in nearby health facilities to overcome barriers to access skilled birth attendants [30]. Even with these interventions, the rate of stillbirth remains high due to the low utilization of maternal health services [31–33]. Moreover, there is a high rate of fertility, desire for an additional child soon and more than half of pregnancies were occurring within a short duration after preceding childbirth (IPI < 24 months) [34]. These situations may aggravate the risk of stillbirth, as it is a pregnancy and childbirth-related issue.

Ethiopia was placed together with those countries which are at risk of missing the target set for Every Newborn Action Plan (below 12 per 1000 total births), and acceleration of current interventions by a factor of 3–5 times are suggested to achieve the target by 2030 [6]. From existing literature, we understood that the relationship between IPI and stillbirth is understudied. World Health Organization (WHO) called for further studies to elucidate the temporal relationship between IPI and its adverse outcomes including stillbirth to strengthen recommendations on pregnancy spacing [23]. A finding from Ethiopian highlighted that a birth interval > 36 months (IPI > 27 months) was found to be protective, from stillbirth [11]. However, that study was not conducted with the purpose of estimating the effect of IPI on stillbirth, and its method for ascertainment of IPI introduced the possibility of misclassification. Thus, we conducted a prospective cohort study to fill these gaps and make the evidence stronger for decision-making. There are feasible interventions to increase IPI to normal (interval with minimum risk of stillbirth) if short IPIs have a temporal relationship with stillbirth. One of these interventions is increasing the utilization of modern contraceptive methods, which can be delivered at community and health facility level, even with lower-level community health works (for example, health extension workers in the Ethiopian context). Concerning this, we hypothesized that IPIs < 18 and 18–23 months have an effect on stillbirth as compared to IPI 24–60 months. Therefore, this study aimed to assess the effect of IPI on stillbirth in urban South Ethiopia. Moreover, we aimed to estimate the incidence of stillbirth in the study setting. The findings contribute to decreasing the stillbirth rate via guiding interventions to achieve Every Newborn Action Plan at the national level and improve pregnancy outcomes.

## Methods

### Study design and setting

This study was a community-based prospective open cohort study. An open cohort study is also called a dynamic cohort, in which study participants can be added or leave from the cohort over time. The study was conducted in the Hadiya zone from July 08/2019-September 30/2020. Hadiya zone is one of the zones in the Southern Nations, Nationalities, and Peoples Region (SNNPR) of Ethiopia, which is located at 232Km far from the capital city, Addis Ababa, and 194 km from the regional capital, Hawassa. The zone has one general hospital, three primary hospitals, 62 health centers and 311 health posts that offer health services for the community [Hadiya Zone Health Bureau report-Unpublished]. In this study, five urban settings (Hossana, Shone, Gimbichu, Jajura and Homecho) which consist of a total of 18 kebeles (lowest administrative units in Ethiopia) were included [35].

### Participants

This study was conducted among pregnant women who had a live birth, during the most recent childbirth, from July 1/2014 onwards in five urban settings, in the Hadiya zone, South Ethiopia. House-to-house visits were done to identify and register pregnant women. During the recruitment, study participants were included in the study based on the eligibility criteria for the exposure variable (IPI). The inclusion criteria were women who: were pregnant at the time of recruitment, had a live birth during the most recent childbirth, were able to recall the date of their last childbirth. Women who had a prior live birth > 60 months earlier, had a recent stillbirth, had a recent abortion and did not show a willingness to be followed were excluded. Then eligible pregnant women were enrolled at the end of 1st trimester (after 12 weeks of gestation) of confirmed pregnancy. This was done every 3 months, for a total of 9 months. An enrolment was done from July 08/2019 to March 30/2020 by trained midwives and the enrolled pregnant women followed until September 30/2020. A total of 2578 pregnant women were enrolled in this study. Of them, 1273 were exposed groups; 769 had IPI < 18 months and 504 had IPI 18–23 months. The remained 1305 were unexposed group (IPI 24–60 months) [35].

### Variables

#### Outcome variable

The dependent/outcome variable was stillbirth (both macerated and fresh stillbirths).

#### Exposure variable

The exposure variable was inter-pregnancy interval.

According to the WHO, inter-pregnancy interval (also known as birth to pregnancy interval) is defined as a time elapsed from live birth to subsequent conception or a woman’s last menstrual period (LMP). According to the WHO, IPI < 24 months is generally defined as short [23]. For this study, we operationally define IPI < 18 months as short, IPI 18–23 months as moderately short and IPI 24–60 months as normal.

#### Confounding variables

The potential confounding variables were maternal age, education, occupation, age at first child birth, parity and pregnancy intention.

### Data sources

A questionnaire was prepared in the English language from existing related literature (published articles and Ethiopia Demographic and Health Surveys (EDHS)) based on the study objectives [11, 22, 26, 34]. The English version was translated to the Amharic version by two native speakers of the Amharic language (one was public health and the other was English language and literature in the profession). Then back translation to English was done by another two individuals who could speak English (again one was from public health and the other from English language and literature). Individuals involved in translations were those who knew the local terminologies for some expressions. The final questionnaire was prepared by involving both groups (translators) after resolving inconsistencies via discussion for some meanings and terminologies. The tool was piloted on 50 pregnant women in Durame town where the actual study population is culturally related. Amendment was made by the investigators. Data was collected by ten trained midwives and supervised by five public health professionals. Data collectors were those who speak both Amharic and local languages (Hadiyisa) to clarify when difficulty in listening to Amharic happened. The training was given for 2 days on the concepts of the questionnaire related to the objectives. Roleplay was made during training on how to approach study participants ethically and make interviews consistently without disrupting the concepts. Comments were given by data collectors, supervisors and principal investigator immediately upon completion of the roleplay. Baseline data about the main exposure variable and other socio-demographic and reproductive characteristics were collected at the household level during enrolment via face-to-face interviews. The data collectors at each health facility were assigned and the list of participants was given for each of them. Outcome (stillbirth) was collected during labor and delivery. In cases when data collectors were not around (e.g. night), data were filled by the informed birth attendant and from the client chart [35].

### Measurement

#### Outcome ascertainment

The outcome variable (stillbirth) was ascertained during labor and delivery as if no sign of life such as spontaneous breathing, heartbeat and voluntary movement. A fetus with skin darkening, redness, peeling, and breakdown was diagnosed as prepartum (macerated) stillbirth. If a fetus lacks such skin and soft-tissue changes, it was diagnosed as intrapartum (fresh) stillbirth. Both macerated and fresh were grouped as stillbirth and categorized as a dichotomous variable (1 = Stillbirth, 0 = live birth).

#### Exposure ascertainment

The exposure variable (inter-pregnancy interval (IPI)) was ascertained by asking women the date of the most recent childbirth and the last menstrual period. By subtracting the date of recent childbirth from the date of last menstrual period (LMP) we could get IPI. For women who had difficulty in recalling the date of LMP, Ultrasound was used to estimate gestational age. By subtracting the duration of gestation, we have got the date of LMP and calculated IPI [23]. To be in line with the WHO recommendation, women with IPIs < 24 months were categorized as an exposed group and IPI 24–60 months as an unexposed group [23]. We further categorized IPI < 24 months as IPI < 18 and 18–23 months, both are the exposed groups. Then, we compared the risk of stillbirth in these groups with that of the unexposed group (IPI 24–60 months).

#### Confounder ascertainment

Confounder variables are those variables that have an association with exposure (IPI) and outcome (stillbirth). These confounders were identified by prior knowledge and from literature (Additional Fig. 1). The potential confounders were ascertained as follow: reported age at interview was measured in completed years. Educational status was measured as no formal schooling, primary education (1st – 8th grade), secondary education (9th - 12th grade) and higher education (>12th grade or certificate, diploma and above). Occupation was measured by asking women the main occupation that they routinely do. Women who were working as a formal employee in any organization (governmental or non-governmental) were categorized as formally employed and those who were merchant/vendor, farmer and housewife were categorized as formally unemployed or others. Age at first childbirth was measured as reported age in completed years when a woman begins childbearing for the first time, irrespective of survival status. Parity was measured as the number of times a woman gives birth, irrespective of the outcomes of birth (live birth or stillbirth). Pregnancy intention was measured as whether a woman has the intention to be pregnant or not at the time of conception.

### Statistical analysis

Collected data were edited and coded manually before entry. Data entry template was prepared in Epi-data version 3.1 software. Then, data were entered by an individual who has experience in Epi-data and exported to SPSS version 20 software for further exploration using frequency tables and visualizing graphically to identify outliers, missing values and transformation. After exporting, data consistency was checked by using the original questionnaire for the responses using participants’ code numbers. After cleaning and making data ready, the data were exported to R version 4.0.5. Descriptive statistics such as frequency and cross-tabulation were done. Mean and standard deviations were calculated for continuous variables. Frequencies and percentages were calculated for categorical variables and discreet continuous variables. For missing data (rare in this study) a complete case analysis approach was used [35]. A generalized linear model (GLM) for binary outcome was applied for the regression analysis. The exposure (IPI) and its potential confounding variables (age, education, occupation, parity, age at first childbirth, and pregnancy intention) were entered in the multivariable model for the adjustment. The confounders were selected based on expert knowledge and evidence from the literature. Multicollinearity between the independent continuous variables, age and age at first childbirth, was assessed using variance inflation factor (VIF). The maximum VIF value was 1.12, which is close to 1 or less than 10, suggesting there was no multicollinearity problem. Thus, both variables retained in the adjusted model. Hosmer and Lemeshow goodness of fit statistics was done and the model was found to be a good fit (*P* = 0.76). The results were interpreted using RR as an effect measure. Attributable fraction (AF) was calculated from adjusted RR to estimate the impact of public health of the exposure (i.e. to what extent the occurrence of stillbirth among the exposed group could be prevented by removing the exposure). Population attributable fraction (PAF) was also calculated to estimate the impact of exposure in the study population (i.e. to what extent the occurrence of stillbirth in the population could be prevented by removing the exposure). AF was calculated by the formula; AF = [(RR-1)/RR]*100. PAF was calculated as; PAF=Pr(exposed/disease)*[(RR-1)/RR] = Pc*AF [36, 37]. Where; RR is the adjusted relative risk and Pc is the percentage of cases exposed (i.e. prevalence of exposure among the cases (stillbirth)). IPI 24–60 months (unexposed group) was used as reference category to estimate the RR and AF of the exposure groups (IPI < 18 and 18–23 months) on stillbirth. A Kaplan-Meir curve was done to assess whether there are differences in cumulative incidence of stillbirth among different categories of IPI (IPI < 18, 18–24 and 24–60 months). A log-rank test was used to assess statistically significant differences among the categories of IPIs. The curve was produced by considering unrecoded IPI as a time variable, recoded IPIs (IPI < 18, 18–23 and 24–60 months) as a factor variable and the stillbirth as a status variable (binary outcome) in the survival analysis model.

## Results

### Cohort profiles

**Fig. 1:**
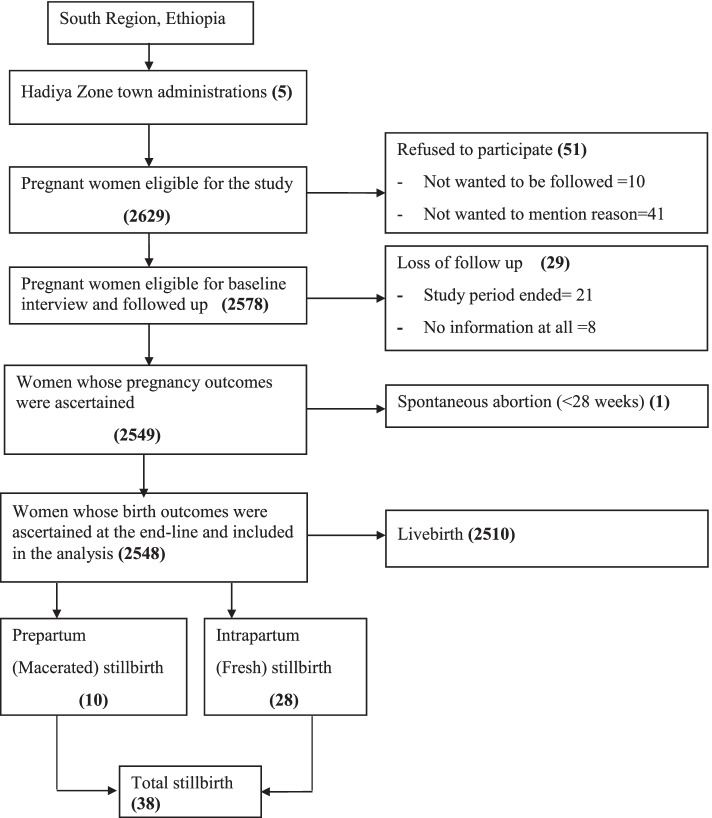
Flow-diagram of the overall study process in urban South Ethiopia, July 2019–September 2020

A total of 2578 participants followed-up until delivery. Of these, 29(1%) of them were lost of follow-up (21 due to end of the study period, 8 no information at all including via phone calling) and their pregnancy outcomes could not be ascertained. Of 29 lost of follow-up, 14 were from exposed and 15 from unexposed groups. The pregnancy outcomes were ascertained for the remained 2549 study participants. One woman had spontaneous abortion before 28 weeks of gestation. Hence she was not followed-up any more. The final analysis was done for the remained 2548 study participants (Fig. 1).

### Stillbirth rate

Of 2548 pregnant women for whom the birth outcomes were ascertained, 38 of them had a stillbirth, yielding a stillbirth rate of 15 per 1000 total births, 95%CI (11, 20%). More specifically, 10 were antepartum (macerated) stillbirths, which accounts for 4 per 1000 total births and 28 were intrapartum (fresh) stillbirths, which accounts for 11 per 1000 total births.

### Socio-demographic and reproductive characteristics of the participants

In this study, missing data were observed for some variables. The missed variables were: age (8), age at first childbirth (5) and parity (5). Missing data were not related to outcome (Stillbirth) since the missed variables were collected at baseline before the occurrence of the outcome.

The mean age of women was 27.5 ± 3.5 years. The age ranges from 20 to 42 years. A higher proportion of the participants were in a primary education level (1st – 8th grades), 1069 (42%).

The mean age at first childbirth was 21.4 ± 2.7 years. A higher proportion of the participants had IPI of 24–60 months, 1290(51%). The incidence of stillbirth was higher among those with IPI < 18 months, 22(58%) than other intervals (Table 1).

**Table 1:** Socio-demographic and reproductive characteristics of the participants in urban South Ethiopia, July 2019–September 2020

Variables	Stillbirth ***N*** = 38	Live birth ***N*** = 2510	Total (%) ***N*** = 2548	X^**2**^ (***P***-value)
	Number (%)	Number (%)		
**Inter-pregnancy interval**				
< 18 months	22 (58)	732 (29)	754 (29)	14.9 (0.001)
18–23 months	4 (10)	500 (20)	504 (20)	
24–60 months	12 (32)	1278 (51)	1290 (51)	
**Education**				
No formal education	6 (16)	495 (20)	501 (20)	1.7 (0.65)
Primary	14 (37)	1055 (42)	1069 (42)	
Secondary	11 (29)	530 (21)	541 (21)	
Higher	7 (18)	430 (17)	437 (17)	
**Occupation**				
Formally employed	8 (21)	399 (16)	407 (16)	0.74 (0.39)
Others	30 (79)	2111 (84)	2141 (84)	
**Parity**				
1–2	28 (74)	1701 (68)	1729 (68)	3.1 (0.22)
3–4	5 (13)	597 (24)	602 (24)	
≥ 5	5 (13)	207 (8)	212 (8)	
**Pregnancy intention**				
Intended	21 (55)	1533 (61)	1554 (61)	0.53 (0.47)
Unintended	17 (45)	977 (39)	994 (39)	
**Methods of estimation of gestational age**				
Last menstrual period	33 (87)	2307 (92)	2340 (92)	1.28 (0.26)
Ultrasound	5 (13)	203 (8)	208 (8)	
**Study site**				
Hossana	17 (48)	961 (38)	978 (38)	4.51 (0.34)
Homecho	5 (13)	423 (17)	428 (17)	
Jajura	0 (0)	200 (8)	200 (8)	
Gimbichu	5 (13)	365 (15)	370 (15)	
Shone	11 (29)	561 (22)	572 (22)	
**Continuous variables**				
Age in years	26.9 (4.1)	27.5 (3.5)	27.5 (3.5)	–
Age at first childbirth in years	21.4 (2.7)	21.4 (2.7)	21.4 (2.7)	–

Others: Merchant/vender/farmer/housewife. **--**: not applicable

Data were missed for parity (5), age (8) and age at first childbirth (5)

For continuous variables ‘mean (standard deviation)’ were estimated for each column

### Effect of inter-pregnancy interval on stillbirth

Adjusting for potential confounding variables (age, education, occupation, age at first childbirth, parity and pregnancy intention), in the multivariable model, IPI was found to be an independent risk factor of stillbirth (adjusted RR = 3.55) with a 95% confidence level (1.64, 7.68) and *P* < 0.05. According to this finding, risk of stillbirth was nearly four times higher for women who had IPI < 18 months than those with 24–60 months. IPI 18–23 months have shown no association with stillbirth as compared to IPI 24–60 months (Table 2).

**Table 2:** Multivariable generalized linear model for the effect of inter-pregnancy interval on stillbirth in urban South Ethiopia, July 2019–September 2020

Exposure variable	Crude RR, 95%CI	Adjusted RR, 95%CI	***P***-value	AF (95%CI)	PAF (95%CI)
**Inter-pregnancy interval**					
< 18 months	3.14 (1.56, 6.30)	3.55 (1.64, 7.68)	0.001	72% (39, 87%)	42% (23, 50%)
18–23 months	0.85 (0.28, 2.63)	0.92 (0.30, 2.87)	0.89	–	–
24–60 months	1	1		1	1

RR adjusted for age, education, occupation, parity, age at first childbirth and pregnancy intention

Keys: *RR* Relative risk, *CI* Confidence interval, *AF* Attributable fraction, *PAF* Population attributable fraction. 1 = reference category

The Kaplan-Meier curve also indicated that there are differences in the cumulative incidence of stillbirth across the IPIs categories (log-rank *P* < 0.001). The cumulative hazard function has shown that the cumulative incidence of stillbirth was increasing or higher for the IPI < 18 months and it was relatively lower in the IPI 18–23 and 24–60 months (Additional Fig. 2).

### Sensitivity analysis

We did a sensitivity analysis to check whether recalling dates of LMP and last childbirth misclassified IPI by reducing 1 month and increasing 1 month from the cutoffs for IPI (24 months). The result of sensitivity analysis indicates that, in both cases, it did not affect the association and conclusion. When we increase by 1 month (RR = 3.13, 95%CI: 1.56, 6.28) and when we decrease by 1 month (RR = 3.09, 95%CI: 1.56, 6.10). That means the presence of misclassification (in case it exists) did not affect the observed estimate and the conclusion. The misclassification, if it exists, would be non-differential.

When participants disappear from either exposed or unexposed group or both (loss of follow-up (LOFU)) might bias estimates. We used phone calling to get information. We also have estimated the impact of LOFU by four assumptions: firstly, if all LOFU developed the outcome, the RR = 2.27, which is within the 95%CI range (1.56, 6.30) when complete case analysis was considered. Secondly, if all LOFU did not develop the outcome, RR = 3.12, which is similarly within the 95% CI range and almost equivalent to RR = 3.14 when complete case analysis was applied. Thirdly, if all exposed developed an outcome but all unexposed did not (worst case scenario), RR = 5.10 which is within 95%CI when complete case analysis was used. All three assumptions indicate the impact of LOFU was minimal. Fourthly, if all unexposed develop an outcome but all exposed did not (best case scenario), RR = 1.38 (diluted towards null) which is outside the range of 95%CI when complete case analysis was done. Finally, loss to follow up in our study was 1%, which is less than recommended < 5%, or not more than 20% [38]. Thus, we can infer the estimates for the target population with minimal cautions with this rule of thumb.

We have also conducted a sensitivity analysis to evaluate whether the outcome (stillbirth) is affected by the estimation methods (LMP and Ultrasound) and study sites. We investigated whether method of estimation of GA and study site could have confounded the association between IPI and stillbirth but found no evidence for this (95% confidence intervals for estimates of associations between each of these covariates and stillbirth included the null values), so we excluded them from our model.

We have also done a sensitivity analysis to check whether considering age and age at first childbirth in the adjusted model have affected the results (effect of IPI on stillbirth) or not by removing one and retaining the other. When removing the age and retaining age at first childbirth, ARR = 0.97, 95% CI: 0.87, 1.10 for age at first childbirth and the ARR = 3.60 for IPI < 18 months. When removing the age at first childbirth and retaining the age, ARR = 0.94, 95% CI: 0.85, 1.05 for age, and the ARR = 3.57 for IPI < 18 month. Retaining both, the ARR = 3.55 for IPI < 18 months. There is a bit differences in decimal values. Since both variables are important, and there is no evidence of multicollinearity problem (VIF < 10), we retained both variables in the model.

## Discussion

In this study, we found an overall rate of stillbirth in the urban settings 15 per 1000 total births. According to the finding, risk of stillbirth was nearly four times (adjusted RR = 3.55, 95%CI: 1.64, 7.68) higher for women who had IPI < 18 months than those with 24–60 months.

In this study, the stillbirth rate was 15 per 1000 total births. It seems relatively low but as this is a pregnancy loss, it should not be regarded as low; rather it suggests the need for policy actions because of long-lasting psychological and social impacts on the mother and families. The rate was still higher than the target set for Every Newborn Action Plan. The result is in agreement with the results of others in Ethiopia that reported 14.1 [39], 13.5 [40] and 18.8 [41] per 1000 births; all were community-based prospective cohort studies. The result was lower than the other studies from the Amhara regions, northwest Ethiopia; 28 [42] and 23.4 [43] per 1000 births. The differences might be explained by variation in geographic location, settings, access to health facilities, outcome ascertainment and eligibility criteria used. Our study was in an urban community but those studies in the Amhara region included both rural and urban. The stillbirth rate is usually higher in a rural community than in an urban [34]. Population characteristics and access to maternal and child health services, together with topography that affect transportations during labor emergencies and accessing obstetric care could be another possible reason [32, 33, 44]. We set inclusion criteria according to the definition for IPI. This might result in underestimation of the rate in our study. For instance, we excluded women with a recent history of stillbirth, which is a known risk factor for subsequent stillbirth [9]. On the other side, the rate in our study was also lower than reports from health facility-based studies elsewhere in Ethiopia; 92 in Yirgalem [10], 36.8 in Axum [13], 71 in Gondar [21], 80 in Jimma [45], 36 in Tigray [46] per 1000 births. It was also lower than those from Tanzania, 35 [2] and Ghana, 34 [19] per 1000 births. This is due to the fact that health facility-based studies over-represent the outcome, as they include a high number of referral cases from rural and neighbors that increase the report. Including referral cases might increase the stillbirth rate, as delays in arrival and receiving care contribute to stillbirth [14, 17]. In contrary, the rate of stillbirth in our study was much higher than that in the North shoa zone, Ethiopia, which was 5.9 per 1000 births [47]. This significant variation might probably be due to the emphasis given to improving maternal and child health care by non-governmental and governmental organizations in the North shoa zone.

Future research on the stillbirth rate needs to focus on the population-based prospective follow-up studies, separately for urban and rural, than health facility-based surveys, as this exaggerates the true incidence of stillbirth due to referrals.

In this study, the evidence suggests that an IPI under 18 months increases the risk of stillbirth after livebirth relative to an IPI of 24–60 months. The result indicated that about 72% of stillbirth among the exposed group (IPI < 18 months) was attributed to the IPI < 18 months. This could be prevented if the IPI under 18 months did not occur in this group namely if the IPI were at least 18 months or longer. Similarly, about 42% of stillbirth in the study population was attributed to the IPI < 18 months, which could be prevented with the removal of IPI under 18 months. This could probably be due to the hypothesis that adequately spacing pregnancies help the uterine wall to recover from the abnormal process of remodeling of endometrial vessels, incomplete healing of uterine scars and nutritional depletion [48, 49]. Inadequate spacing affects the time to restore the physiological needs for the fetus due to placental insufficiency that causes growth restriction [50]. In supporting this, a study from Bale zone, Ethiopia highlighted higher odds of stillbirth when IPI was < 15 months [22]. The other from the Amhara region, Ethiopia has reported that birth interval above 36 months (approximately equivalent to IPI > 27 months, subtracting 9 months gestational age) was protective [11].

IPI is a modifiable risk factor of stillbirth. IPI under 18 months can be prevented by using family planning methods consistently since one of the big pillars of family planning is spacing pregnancies. Spacing pregnancy to 24–60 months can be achieved by consistent use of modern contraceptive methods, which are cost-free and can be implemented even by consulting primary health care providers such as health extension workers in the Ethiopian context. However, there are several issues related to contraceptive service utilization that need to be addressed. Some of which are lack of knowledge, myths, misconception, health concerns (fear of side-effects), lack of support from husband/partner and lack of access [51, 52]. Giving health education to couples and the community at large might help to overcome these barriers to the use of contraceptive methods. Research is needed to assess whether reducing those barriers of contraceptive utilization increase inter-pregnancy interval to at least 18 months and subsequently reduce the incidence and risk of stillbirth or not. IPI after preceding stillbirth needs research to assess the adequate duration of IPI that reduces the risk of stillbirth in a subsequent pregnancy to make a recommendation.

This study might have limitations related to some potential biases, as it depends on retrospective ascertainment of the dates of the last menstrual period and the last childbirth. However, we have attempted to reduce those biases by early recognizing the possibility during the design stage as follow:

Selection bias: In our study, determining gestational age by Ultrasound was not feasible for all study participants and also it was not available in most health facilities in Ethiopia. Because of these reasons determining gestational age by LMP was an alternative option. On the other side, excluding women who did not remember LMP leads to selection bias. Thus, for those women who could not be able to recall LMP due to breastfeeding, contraceptive method use, and other reasons, we used Ultrasound as a solution to minimize selection bias. We included pregnant women during the 9 months enrollment so that this population-level study also helps to minimize selection bias. Due to cost constraints for laboratory tests, enrolment was made after the first-trimester because first-trimester pregnancy was detected mainly via laboratory tests and some women might not know whether they are pregnant (another potential source of selection bias). Hence we enrolled pregnant women who had already confirmed pregnancy or visible pregnancy.

Recall bias: happened when the participants asked the date of preceding childbirth and last menstrual period. In the urban community, it is common to see birth date ceremonies, it was used in addition to the verbal report. An immunization card was also used where available which helps to see the date that immunization was initiated, usually initiated at 45 days of delivery in addition to those vaccines given at birth. Family members such as husband, mother-in-law and others who exactly remember the date of recent childbirth were used to support the women in recalling. Furthermore, we limited the time of the most recent childbirth date, within the last 5 years from the date of data collection to minimize bias related to recall.

Despite the limitations, this study has strong sides: firstly, it was community-based hence reduce selection bias as compared to health facility-based studies. Secondly, it was a prospective cohort study design that is strong in elucidating temporal relationships and reporting incidence than other observational studies. Thus, considering the aforementioned caveats, the findings can be generalized for the urban community with similar contexts in Ethiopia.

## Conclusions

This study found out that, inter-pregnancy interval under 18 months increases the risk of stillbirth in this population in urban South Ethiopia. Inter-pregnancy interval is a modifiable risk factor. Thus, interventions targeted to factors contributing to short inter-pregnancy interval could be helpful to reducing the risk of stillbirth. Improving contraceptive utilization in the community could be one of these interventions. Therefore, couples should be informed about how long to wait until the subsequent pregnancy and the risks of inadequately spaced pregnancies like stillbirth.

## Supplementary Information

**Additional file 1: Figure S1.** Theoretical frame work for the effect of inter-pregnancy interval on stillbirth, and potential confounding variables.

**Additional file 2: Figure S2.** Kaplan–Meier survival curve showing differences in the cumulative incidences of stillbirth for the categories of inter-pregnancy interval (Log-rank *P* < 0.001).

## Data Availability

The raw materials that support the conclusions of this research will be available to researchers, who need the data to use for non-commercial purposes through requesting the corresponding author.

## Abbreviations

AF: Attributable fraction
EDHS: Ethiopia Demographic and Health Survey
IPI: Inter-pregnancy interval
PAF: Population attributable fraction
WHO: World Health Organization
