# Supplementary material for: The effect of inter-pregnancy interval on stillbirth in urban South Ethiopia: a community-based prospective cohort study
Source: BMC Pregnancy Childbirth. 2021 Dec 29;21:847. doi: 10.1186/s12884-021-04325-z (PMC8715581; doi:10.1186/s12884-021-04325-z)
Supplement: Supplementary file 1 — Additional file 1: Figure S1. Theoretical frame work for the effect of inter-pregnancy interval on stillbirth, and potential confounding variables. [file 12884_2021_4325_MOESM1_ESM.docx]

Additional figure 1.

| **Outcome variable:**   - Stillbirth   **Exposure variable:**   - Inter-pregnancy interval   **Potential confounding variables:**   - Age - Age at first child birth - Parity - Occupation - Education - Pregnancy intention   Additional figure 1. Theoretical frame work for the effect of inter-pregnancy interval on stillbirth, and potential confounding variables. |
| --- |
