# Supplementary material for: The effect of inter-pregnancy interval on stillbirth in urban South Ethiopia: a community-based prospective cohort study
Source: BMC Pregnancy Childbirth. 2021 Dec 29;21:847. doi: 10.1186/s12884-021-04325-z (PMC8715581; doi:10.1186/s12884-021-04325-z)
Supplement: Supplementary file 2 — Additional file 2: Figure S2. Kaplan–Meier survival curve showing differences in the cumulative incidences of stillbirth for the categories of inter-pregnancy interval (Log-rank P < 0.001). [file 12884_2021_4325_MOESM2_ESM.docx]

Additional figure 2

| 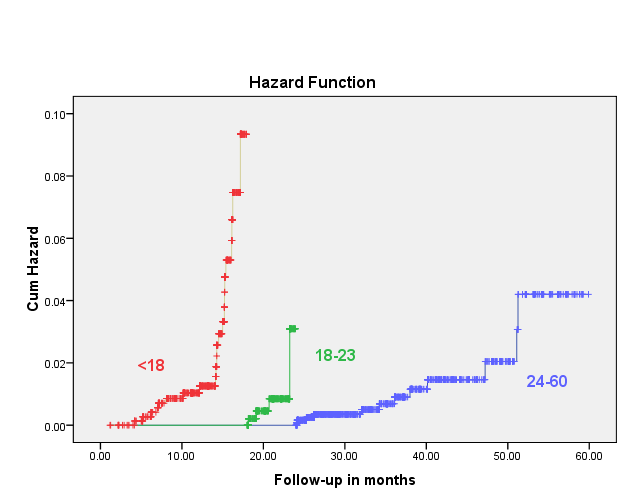  Additional figure 2. Kaplan–Meier survival curve showing differences in the cumulative incidences of stillbirth for the categories of inter-pregnancy interval (Log-rank P <0.001). |
| --- |
